# Social Engagement and Sense of Community Among Older Adults: The Role of Socioeconomic Status and Race

**DOI:** 10.1093/geroni/igaf122.505

**Published:** 2025-12-31

**Authors:** Bryant Carlson

**Affiliations:** Oregon Health & Science University - Portland State University School of Public Health, Oregon, United States

## Abstract

Previous research suggests that as levels of neighborhood socioeconomic and racial heterogeneity increase, rates of social engagement and sense of community (SOC) often decrease, but the extent to which this trend persists for older adults in socioeconomically and racially diverse communities remains unclear. This study estimated the relationship of engagement in social activities with SOC and its components among Black or African American, Hispanic or Latino, and White older adults. Participants included 204 older adults aged 60 years or older. Social engagement was assessed with eight questions, and the Brief Sense of Community Scale (BSCS) was used to assess total SOC and components of SOC (e.g., need fulfillment, group membership, influence, and emotional connection). Results were analyzed using confirmatory factor analysis and hierarchical linear regression. Engagement in social activities had a positive effect on SOC, but this relationship varied by socioeconomic and racial group. Specifically, engagement in social activities had a positive relationship with SOC for White and higher socioeconomic level older adults, but the strength of this relationship attenuated considerably for Black or African American, Hispanic or Latino, and lower socioeconomic level older adults. While these findings validate the importance of engagement in social activities for increasing SOC in older adults, the effect observed for neighborhood socioeconomic and racial heterogeneity poses challenges for SOC in diverse neighborhoods and communities, reinforcing enduring tensions between community cohesion and diversity.

